# The Treatment of Fractures from a Common-Sense Point of View

**Published:** 1907-10-05

**Authors:** 


### Points in Surgery.

### THE TREATMENT OF FRACTURES FROM A COMMON-SENSE POINT OF VIEW.

### VII. FRACTURES OF THE UPPER END OF THE HUMERUS.

It is no exaggeration to say that the exact diagnosis of the condition of affairs after a severe injury to the upper end of the humerus may be one of the most perplexing problems in surgery. Fractures in this situation are divided by the writers of text-books into (1) fractures of the surgical neck, by which is meant a transverse fracture of the bone about one to two inches below the head; (2) fracture of the anatomical neck, in which the rounded head or articular surface is separated from the shaft; (3) fracture at the morphological neck or separation of the upper epiphysis.

#### Fracture of the Surgical Neck.

Fracture of the surgical neck is the most frequent of these. It is usually the result of indirect violence, and is brought about by the same circumstances that produce fracture of the clavicle, that is, the transmission of the weight of the falling body to the shoulder through the outstretched hand. It is not an uncommon accident in the hunting field. As in all fractures of a long bone in a limb the patient will complain of pain, and loss of use of the limb. On inspection it will be found that there is unnatural mobility of the arm, that is to say that the limb can be passively moved, although the head of the humerus is held firmly in position. In many cases crepitus cannot be obtained, either because the fragments are completely separated from one another or because the pain is too great to allow of the necessary manipulation. In the latter case strenuous efforts to obtain it should never be made; they are not only brutal but they are unnecessary, since to suppose that the obtaining of crepitus is absolutely essential in making a diagnosis of fracture is a relic of the prehistoric days of surgery. The amount of displacement found varies enormously; in some cases the ends of the bone are hardly separated, while in others the fractured ends may not be in apposition at all. The upper fragment is most often found to be occupying its normal position, as one would expect from a consideration of the anatomy of the part. The pull exerted by the strong subclavius muscle, tending to rotate the bone inwards, is just about counteracted by the three muscles inserted into the great tuberosity, the supra-spinatus, the infra-spinatus, and the teres minor, which have an exactly opposite action. The lower fragment is pulled upwards and inwards by the muscles attached to the bicipital groove, so that it lies in front of the upper fragment, and its upper end can be felt below the coracoid process. In rarer cases, however, it lies behind. The diagnosis may be more or less obvious or it may be obscure. This is particularly the case when the lower fragment is firmly impacted in the upper, so that one of the main diagnostic features of fracture of a long bone, loss of use of the affected limb, is absent. The writer remembers one such case. A man fell from a ladder while painting a house, and sustained injuries to his head and his right shoulder. He was not in very great pain, and went home without seeing a doctor. The pain persisted for some days, and he sought relief at a hospital, where he was seen by the house surgeon. On examination it was found that he could move his arm freely in all directions, and there was no shortening. He was accordingly treated with goulard and opium fomentations. Two weeks later a swelling was noticed in the upper part of the humerus, and a radiograph was taken. It was then found that there had been a slightly oblique fracture of the surgical neck, and that the lower fragment was firmly impacted in the upper. The swelling was, of course, due to callus. A perfectly satisfactory result was eventually obtained. This case is quoted to show the ease with which a fracture in this neighbourhood may be missed altogether unless a radiograph is taken. In the first article of this series great stress was laid on the importance of doing this in all cases in which a bone may have been injured. The whole object of the surgeon in the treatment of this injury is to attempt to give the patient a useful arm. To this end an anæsthetic should be given in all cases and the ends of the fragments manipulated into position if possible. If there is impaction, without much shortening, and the limb is in its normal line, there is no indication to disimpact. A pad should be placed in the axilla to prevent the upper end of the lower fragment being pulled inwards, and the whole arm bound to the side with a bandage. A poroplastic cap may be cut out and fitted over the shoulder; it is kept in position by tapes, which are attached to holes in its edges and tied round the body. Whether the poroplastic cap serves any more active purpose than to persuade the patient that something particular is being done for him is an open question. It is difficult to see how it could have any action in keeping the fragments in position. The most it can do is to protect the shoulder from further injury. Massage and passive movement are of extreme importance in these cases, and must be begun early if permanent limitation of movement, owing to adhesions in the shoulder joint, is to be avoided. One more practical piece of advice. In hospitals these cases are treated as out-patients from necessity, owing to pressure on beds, but it will be found, in the case of private patients, that rest in bed for the first three or four days at the beginning of treatment is of great help to surgeon and to patient, because it diminishes the pain and muscular spasm which is associated with this injury. So far it has been supposed that reduction by manipulation under an anæsthetic has been successful. But there are cases in which the greatest amount of skill and perseverance is unable to effect this, or in which the displacement recurs after reduction. What is to be done then? The surgeon has the choice of two alternatives. One is to put the patient to bed and apply extension to the arm. This sounds an easy thing to do, but in practice it is quite the reverse. A stirrup is fixed to the arm with strapping, and the extension is applied by means of a weight, which passes over a pulley at the side of the bed. The arm is kept somewhat away from the side of the body. Counter extension is applied by means of a binder, which passes round the body under the axilla, and is attached to the bed on the opposite side. It may be said at once that the method of applying extension by allowing the elbow to hang free, as advised in the text-books, is quite useless, and is foredoomed to failure. The other alternative is to perform an open operation and to fix the over-riding fragments in position with silver wire. It is advisable to lay the facts of the case fairly before the patient and to offer him the choice of operation; if, after this, he refuses, the responsibility for a bad result lies more directly with him than it does with the surgeon.

### Fracture of the Surgical Neck, Complicated by Dislocation.

Sometimes fracture of the surgical neck of the humerus is combined with dislocation of the shoulder joint, especially if the violence producing the injury is extreme. The mistake that is most often made in this case is to observe the presence of a dislocation but to overlook the fracture, a mistake that would be impossible if all doubtful cases were radiographed on principle. The condition is very difficult to treat. An attempt should be made to reduce the upper fragment under an anæsthetic. This is rarely successful, because the upper fragment is so short that a firm hold of it cannot be obtained, and no leverage can be exerted to force the head of the bone back through the rent in the capsule of the joint. In the older text-books of surgery one is advised to take measures for securing union of the humerus, and, when this has occurred, to attempt to reduce the dislocation by manipulation. It need hardly be pointed out that this method has obvious disadvantages, not the least of which is that any attempt to reduce a dislocation at so late a date after the accident is more likely to end in a second fracture of the humerus than in successful reduction. The only correct treatment in such a case is to explain the exact state of affairs to the patient and to perform an open operation. In the absence of any indication for immediate operation (such as an effusion of blood, due to hæmorrhage from a torn vessel of any size, that is rapidly increasing), it is better to wait two or three days before undertaking this. During this time the patient is kept in bed, and can recover from the shock of the accident and the part can be rendered thoroughly aseptic. An incision is then made as for opening the shoulder joint, and the ends of the fractured bone are exposed. It may now be possible to get a firm grasp of the upper end and manipulate the head of the bone back through the rent in the capsule. Better leverage can be obtained, if desired, by drilling the bone and inserting a strong hook into the drill hole. When the upper fragment has been reduced, the fractured ends should be united and fixed in position with silver wire; and the rest of the treatment is carried out on the general lines already indicated.

### Fracture of the Anatomical Neck.

Fracture of the anatomical neck of the humerus is a much rarer form of injury. It is generally produced by direct violence in old people. Without a radiograph it is exceedingly difficult to diagnose accurately, since there is generally so much effusion that it is difficult to make out the exact state of affairs. In a typical case there is slight flattening over the deltoid region and about half an inch of shortening in the upper arm, measured from the tip of the acromion process to the external condyle. Sometimes the head of the bone can be felt loose in the shoulder-joint, and the upper end of the lower fragment is drawn up under shelter of the acromion. Here again the correct form of treatment is an open operation, the head of the bone being completely removed. The patient gets surprisingly good movement at the shoulder after this operation. Conservative treatment is likely to give a bad result. If for constitutional reasons it is thought inadvisable to operate, an attempt must be made to manipulate the head in position and to keep it there by bandaging the whole arm to the side with a pad in the axilla. It is most important that massage and passive movement should be begun early.

### Fracture of the Morphological Neck or Separation of the Upper Epiphysis.

Separation of the upper epiphysis is an accident which only occurs before the twentieth year, when the epiphysis unites with the shaft. Clinically it resembles fracture of the surgical neck, the points of difference being the age of the patient (although quite a young patient may sustain a fracture of the surgical neck), and the fact that the line of fracture, as shown by a radiograph, is higher up. Complete displacement of the fragment is rare in this injury because of the close connection of the periosteum to the bone at the epiphyseal line. It is vitally important in these cases to secure extremely accurate coaptation of the fractured ends, otherwise the subsequent growth of bone will be interfered with by the formation of callus along the epiphyseal line. The fragments must therefore be manipulated into position, and then again examined under the $x$-rays to see if the coaptation is perfect. If necessary the manipulations may be carried out under the $x$-rays, so that the effect of the manipulation may be at once appreciated. When satisfactory coaptation has been obtained the rest of the treatment is the same as for fracture of the surgical neck.

